# The Risk of Multiple Myeloma Is Reduced in Metformin Initiators: A Retrospective Cohort Study in Taiwanese Patients with Type 2 Diabetes Mellitus

**DOI:** 10.3390/cancers14225637

**Published:** 2022-11-17

**Authors:** Chin-Hsiao Tseng

**Affiliations:** 1 Department of Internal Medicine, National Taiwan University College of Medicine, Taipei 10051, Taiwan; ccktsh@ms6.hinet.net; 2 Division of Endocrinology and Metabolism, Department of Internal Medicine, National Taiwan University Hospital, Taipei 10002, Taiwan; 3 National Institute of Environmental Health Sciences, Zhunan 35053, Taiwan

**Keywords:** metformin, multiple myeloma, pharmacoepidemiological study, Taiwan, type 2 diabetes mellitus

## Abstract

**Simple Summary:**

Metformin exerts anti-cancer effects but its effect on multiple myeloma requires investigation. This study used the nationwide database of Taiwan’s National Health Insurance to examine whether metformin use in patients with type 2 diabetes mellitus would have a reduced risk of multiple myeloma. Intention-to-treat analyses showed that patients who receive metformin treatment within the first 12 months of prescription of antidiabetic drugs have an approximately 30% lower risk than those who do not. In the per-protocol analyses, patients who adhere to metformin treatment will have an even lower risk reduction of approximately 65%. The findings of this study support an anti-cancer effect of metformin on multiple myeloma and provide a good reason for the recommendation of metformin as the first-line antidiabetic drug for patients with type 2 diabetes mellitus. In patients without contraindications, patients should be advised to maintain on metformin use because of its multiple pleiotropic benefits.

**Abstract:**

Background: Whether metformin might reduce the risk of multiple myeloma (MM) has not been extensively researched in humans. Methods: The study subjects were enrolled from the reimbursement database of Taiwan’s National Health Insurance. A total of 739,553 patients who had a new diagnosis of type 2 diabetes mellitus during 1999–2009 were identified. They were categorized as metformin initiators (metformin (+)) and non-metformin initiators (metformin (−)) based on the prescriptions of antidiabetic drugs that included metformin and did not include metformin within the initial 12 months, respectively. MM incidence was calculated after the initial 12 months of treatment group assignment until 31 December 2011. Hazard ratios based on intention-to-treat (ITT) and per-protocol (PP) approaches were estimated by Cox regression weighted by propensity scores. Results: In the ITT analyses, the respective incidence rates for 497,248 metformin (+) and 242,305 metformin (−) were 9.97 and 14.33 per 100,000 person-years. The hazard ratio that compared metformin (+) to metformin (−) in the ITT analysis was 0.710 (95% confidence interval 0.593–0.850). In the PP analysis, the respective incidence rates were 5.14 and 13.98 per 100,000 person-years, and the hazard ratio was 0.355 (95% confidence interval, 0.270–0.466). The lower risk of MM among metformin (+) was supported by subgroup and sensitivity analyses. Conclusions: Type 2 diabetes patients who are initiated with metformin treatment have a significantly lower risk of MM, especially when they adhere to metformin treatment.

## 1. Introduction

Multiple myeloma (MM) is the second most common hematological malignancy after lymphoma [1]. It accounts for 1% of all cancers and represents approximately 10% of all hematological cancers [2]. MM is characterized by bone marrow plasmacytosis with clinical manifestations of hypercalcemia, renal failure, anemia or lytic bone lesions [2]. Although the etiology remains unknown, it is associated with some gene mutations and linked to diabetes mellitus, metabolic syndrome and obesity [2,3,4,5,6]. Ionizing radiation can also be a risk factor for MM [7]. Most patients develop MM from an asymptomatic premalignant stage called monoclonal gammopathy of undetermined significance (MGUS), which can be present in approximately 5% of the population above the age of 50 [2]. Approximately 1% of the population with MGUS progresses to MM per year [1,2]. Smoldering MM is a more advanced premalignant stage, which progresses to MM at a rate of approximately 10% per year over the first year of diagnosis [1,2]. In the USA, the median age at diagnosis of MM is 69 years, and African Americans have twice the incidence of MM compared to European Americans [1].

The incidence of MM is lower in Asian populations than in westerners [8]. In Taiwan, the average age at the diagnosis of MM is 67.6 years, and the age-adjusted incidence has increased from 1.41 per 100,000 population in 2007 to 1.59 per 100,000 population in 2012 (*p* = 0.01) [8]. On the other hand, the age-standardized incidence in western countries is approximately 5 per 100,000 population [1].

Diabetes mellitus and MM are closely related [4]. An early meta-analysis that included 10 observational studies suggested a non-significantly higher risk of MM while comparing diabetes patients to non-diabetic people with an estimated odds ratio of 1.22 (95% confidence interval: 0.98–1.53, *p* = 0.08) [9]. Another recent meta-analysis that included 13 studies estimated an odds ratio of 1.60 (1.13–2.26, *p* < 0.001) [10]. A recent population-based study published after the latest meta-analysis that used healthcare databases from Ontario, Canada, suggested a significant 15% higher risk of MM in diabetes patients [11]. The estimated incidence was 19.0 per 100,000 non-diabetic people and 25.7 per 100,000 diabetes patients, and the estimated hazard ratio was 1.15 (95% confidence interval: 1.09–1.20, *p* < 0.0001) [11].

Metformin reduces the risk of several types of cancer [12,13,14,15,16]. In our previous study, we also demonstrated a significantly lower risk of non-Hodgkin lymphoma (another blood cancer that is associated with obesity) among metformin users in patients with type 2 diabetes mellitus [17].

In recent years, although a large number of basic research has suggested a promising effect of metformin on the inhibition of the proliferation of MM cells either via 5’ adenosine monophosphate-activated protein kinase (AMPK)-dependent or AMPK-independent mechanisms [18], only a few studies have investigated such an effect in humans. In a large study that included male US military veterans, metformin use was associated with a reduced risk of progression of MGUS to MM [19]. However, this could not be supported by later nested case-control studies conducted in the UK [20,21]. To our knowledge, there has not been any previous human population-based study that investigated whether metformin could be preventive for the development of MM in patients with type 2 diabetes mellitus. In this study, from the nationwide database of Taiwan’s National Health Insurance (NHI), we enrolled patients with a new diagnosis of type 2 diabetes mellitus to compare the risk of MM between metformin initiators (metformin (+)) and non-metformin initiators (metformin (−)).

## 2. Materials and Methods

### 2.1. The Nationwide Database of NHI

Since 1 March 1995, Taiwan has started to implement a nationwide and compulsory healthcare system, the NHI. The coverage rate is very high and includes over 99% of Taiwan’s population. The Bureau of the NHI signs contracts with all hospitals and >93% of all medical settings across the country to provide medical services to the covered insurants. The NHI database contains all information on disease diagnoses, medication prescriptions and clinical procedures being submitted for reimbursement purposes. The database can be used for academic research if the proposal is reviewed and approved by an Ethics Review Board. The present study was reviewed and approved by the Ethics Review Board of the National Health Research Institutes with an approval number of 99274. The database was described in more detail previously [22].

### 2.2. Disease Codes

During the study period, the NHI used the International Classification of Diseases, Ninth Revision, Clinical Modification (ICD-9-CM) as the coding system for disease diagnoses. The disease diagnoses and their corresponding ICD-9-CM codes used in the study are shown in Supplementary Table S1. The accuracy of disease diagnoses in the NHI database has been investigated, which showed moderate to substantial agreements between claim data and medical records, with kappa values ranging from 0.55 to 0.86 [23].

Patients were classified as metformin (+) or metformin (−) based on the prescriptions of antidiabetic drugs after diabetes diagnosis during the initial 12 months as described in our previous studies [17,24,25]. Metformin (+) referred to patients whose prescription during the initial 12 months included metformin. Metformin (−) was assigned to patients who had not been prescribed metformin during the initial 12 months.

### 2.3. Enrollment of Study Subjects

**Figure 1:**
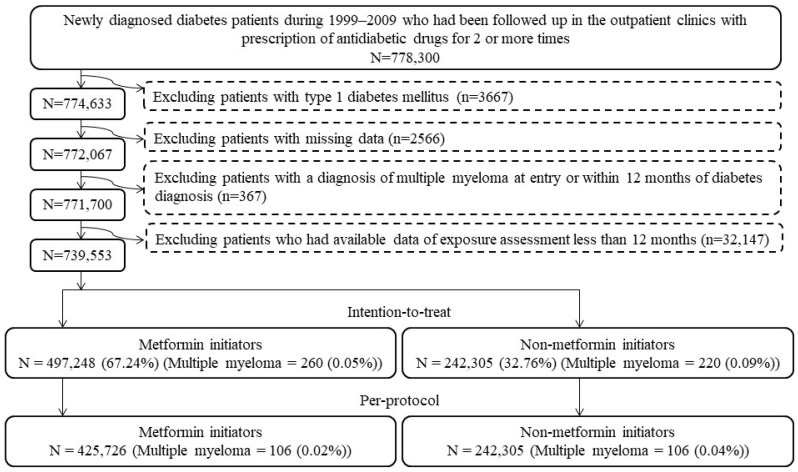
Flowchart presenting the steps followed to enroll metformin initiators and non-metformin initiators for the intention-to-treat and per-protocol analyses in the study.

Figure 1 shows the step-by-step procedures followed in enrolling metformin (+) and metformin (−) patients from the database. A total of 778,300 patients were first identified based on the following two criteria: (1) the patients should have had a new diagnosis of diabetes mellitus from 1999 to 2009 (patients with a diagnosis of diabetes mellitus made during the period from 1995 to 1998 were not included); and (2) they should have been treated at the outpatient clinics with at least two incidences of prescriptions of antidiabetic drugs. We then excluded stepwise the following ineligible patients: (1) patients with a diagnosis of type 1 diabetes mellitus (n = 3667); (2) patients having missing data (n = 2566); (3) patients having been diagnosed with MM before follow-up or within 12 months of diabetes diagnosis (n = 367); and (4) patients who had available data of exposure assessment of less than 12 months (n = 32,147). As a result, 497,248 metformin (+) and 242,305 metformin (−) subjects were used for the intention-to-treat (ITT) analyses, and 425,726 metformin (+) and 242,305 metformin (−) subjects who adhered to the initial assignments were used in the per-protocol (PP) analyses.

### 2.4. Potential Confounders

Potential confounders are shown in Table 1. The “time without antidiabetic drugs after diabetes diagnosis” was defined as the time when the patients were not treated with any antidiabetic drugs after a diabetes diagnosis. Occupation was classified as class I, II, III and IV [17]. Class I included civil servants, teachers, employees of governmental or private businesses, professionals and technicians. Class II included people without a specific employer, self-employed people and seamen. Class III referred to farmers and fishermen. Class IV included low-income families supported by social welfare and veterans. Use of immunosuppressants was defined as continuous use of ≥90 days of corticosteroids, calcineurin inhibitors and/or inosine-5′-monophosphate dehydrogenase inhibitors.

Helicobacter pylori (HP) infection was defined previously by one or two of the following criteria [26]: (1) patients who had received an HP eradication therapy; and (2) patients who had been diagnosed with HP infection.

### 2.5. Statistical Analyses

We used SAS statistical software version 9.4 (SAS Institute, Cary, NC, USA) for statistical analyses and considered *p* < 0.05 as statistically significant.

As a test of balance diagnostics, we calculated the standardized difference for each covariate. A value of standardized difference >10% was viewed as an indicator of potential confounding.

In the ITT analyses, we started to follow the patients after the initial 12-month period used for exposure assessment and ended follow-up at a time until 31 December 2011 when any of the following three events occurred, whichever first, with no exclusion according to switching to or adding other antidiabetic drugs thereafter [17]: the last reimbursement record, MM diagnosis or death. The numerator of incidence was the case number of newly diagnosed MM during the follow-up, and the denominator was the person-years of follow-up.

In the PP analyses, we first excluded patients who did not adhere to the assigned treatment within the initial 12-month period of exposure assessment and then followed the rest for the incidence of MM. We started follow-up after the 12-month period as we have previously done in the ITT analyses. Besides the three events (the last reimbursement record, MM diagnosis or death) to end follow-up at a time until 31 December 2011, follow-up also ended when nonadherence to the assigned treatment occurred, which was defined by the time of addition of metformin in the metformin (−) group, and by the time of addition of non-metformin antidiabetic drugs in the metformin (+) group [17].

We used logistic regression to create propensity scores (PS) from independent variables that included all variables listed in Table 1 plus the date of the start of follow-up. The inclusion of the starting date of follow-up was expected to partly account for some unknown risk factors that might have occurred during the long inclusion period, such as changes in treatment guidelines or the introduction of novel therapeutic drugs. We then estimated hazard ratios and their 95% confidence intervals that compared metformin (+) to metformin (−) by Cox regression constructed with the inverse probability of treatment-weighting using PS. This method for the estimation of PS-weighted hazard ratios is recommended by Austin to reduce the potential confounding by indication because of the differences in baseline characteristics [27].

Age was categorized into two subgroups of <60 and ≥60 years, and subgroup analyses were conducted for each subgroup of age and sex.

To examine the consistency of the findings, we conducted four sensitivity analyses: (1) patients receiving two consecutive prescriptions of metformin spanning a period of more than 6 months were excluded; (2) patients having been treated with incretin-based therapies during follow-up were excluded (the NHI did not reimburse incretin-based therapies until after 1 March 2009); (3) patients having been treated with thiazolidinediones were excluded because thiazolidinediones may cause bone loss and fractures [28] leading to a differential detection rate of MM; and (4) patients with a diagnosis of any cancer other than MM during follow-up were excluded.

## 3. Results

Table 1 shows the baseline characteristics of the study subjects. Metformin (−) and metformin (+) varied in six characteristics with values of standardized difference >10%: time without antidiabetic drugs after diabetes diagnosis, occupation, dyslipidemia, obesity, eye disease and statins.

In the ITT analyses, for metformin (−) and metformin (+) subjects, respectively, the median follow-up time was 6.35 years and 4.81 years. The respective follow-up times in the PP analyses were 2.34 and 4.35 years. The incidence of MM and the hazard ratios comparing metformin (+) to metformin (−) are shown in Table 2. Both the ITT and PP analyses favored a lower risk of MM in the metformin (+) group. The hazard ratio (95% confidence intervals) was 0.710 (0.593–0.850) in the ITT analysis and was 0.355 (0.270–0.466) in the PP analysis.

Table 3 shows the results of the subgroup analyses. It was noted that the lower risk associated with metformin use could be observed in both sexes in both the ITT and the PP analyses. In the analyses with regards to age subgroups, the significantly lower risk associated with metformin use could be seen in the PP analyses with either younger age (<60 years) or older age (≥60 years). However, the lower risk associated with metformin was borderline significant in the ITT analyses in both age subgroups.

As shown in Table 4, all sensitivity analyses supported a lower risk of MM among metformin (+) in either the ITT analyses or the PP analyses.

As observed in Table 2, Table 3 and Table 4, the hazard ratios estimated in the PP analyses showed a more remarkable risk reduction among metformin (+) than those estimated from their corresponding ITT analyses, suggesting that adherence to metformin treatment resulted in more favorable protection.

## 4. Discussion

### 4.1. Main Findings

This population-based observational study first investigated the risk of MM with regard to metformin exposure in an Asian population with type 2 diabetes mellitus. A significant risk reduction of 30% in metformin (+) subjects in the ITT analysis and a risk reduction of 65% in the PP analysis (Table 2) were noted. The risk reduction among metformin (+) was supported by subgroup analyses (Table 3) and sensitivity analyses (Table 4). The risk reduction in metformin (+) was more remarkable in the PP analyses than in the ITT analyses in all analyses (Table 2, Table 3 and Table 4).

### 4.2. Findings in Earlier Studies

The findings of the present study supported a preventive role of metformin in the development of MM, as observed in a previous study conducted on male US military veterans that showed a reduced risk of progression of premyeloma stage to MM [19]. However, the generalizability of the USA study was limited because it was not a population-based study, involved mainly male patients with diabetes (98%), and metformin use was defined as a use of 4 years or longer [19]. The investigators estimated an adjusted hazard ratio of 0.47 (95% confidence interval: 0.25–0.87) [19], which was close to the PS-weighted hazard ratio of 0.425 (95% confidence interval: 0.294–0.616) in the PP analysis for the subgroup of males (Table 3) in our study.

There are two nested case-control studies that were conducted in the UK [20,21]. One showed a null association between metformin use and the incidence of MGUS [20]. In this study, the investigators used a nested case-control study design and selected 4 controls matched on age, sex, practice site and duration of follow-up for each case of incident MGUS [20]. They estimated odds ratios rather than hazard ratios, and the duration of exposure to metformin was not mentioned. Therefore, whether the time of exposure was sufficient for an effect to occur was not known. Though not significant, a 23% lower risk of MGUS (adjusted odds ratio: 0.77, 95% confidence interval: 0.56–1.05) was associated with metformin use.

The second study conducted by the same UK group looked at the progression of MGUS to MM by using a matched case-control study nested within a population-based database of The Health Improvement Network [21]. Among the diabetes patients, there were 11 cases and 127 controls, and the adjusted odds ratio was 1.01 (0.18–5.65) for metformin exposure <24 months and 0.40 (0.08–2.04) for those with metformin exposure >24 months [21]. Though not significant, probably because of the small numbers of cases and controls, an approximately 60% lower risk of progression was observed among patients who had been exposed to metformin for >24 months.

### 4.3. Mechanisms

Although the mechanisms of this clinical benefit of metformin remain to be explored, findings from basic research provide reasonable explanations for the mode of action either through an AMPK-dependent or an AMPK-independent pathway [18]. These may include: (1) the induction of cell cycle arrest and autophagy in MM cells [29]; (2) the inhibition of the HIF-1 pathway of MM leading to growth arrest without inducing apoptosis [30]; (3) the inhibition of MM serum-induced endothelial cell thrombosis by downregulating miR-532 [31]; (4) the inhibition of IL-6 signaling by decreasing IL-6R expression on MM cells [32]; (5) acting as an oxidative phosphorylation inhibitor [33]; (6) the induction of necrosis and apoptosis in MM cells [34]; (7) suppressing glucose-regulated protein 78, an endoplasmic reticulum chaperone with anti-apoptotic properties [35]; and (8) lowering intracellular pH and enhanced cytotoxicity [36].

Additionally, metformin may target obesity (a major risk factor for MM [6]) and the metabolic pathways of MM cells [37,38]. Research has also suggested that metformin may act synergistically with other chemotherapeutic agents to inhibit the growth of MM [38,39,40,41,42].

However, an in vitro and in vivo study showed that metformin might exert an indirect pro-tumorigenic effect on MM by increasing OPN expression in preosteoblasts and thus increasing myeloma cell adherence [43]. Metformin treatment may also induce resistance to the proteasome inhibitor bortezomib in cancer cells [44]. Therefore, the beneficial effect of metformin on MM requires more extensive research.

### 4.4. Implications

This study has some clinical implications. First, the protective effect of metformin against MM, as shown in the present study, together with the known extra bonuses beyond its glucose-lowering effect, such as its anti-cancer, anti-inflammatory, anti-microbial and anti-aging effects [45,46,47,48,49,50,51,52,53,54,55], provide a good reason to recommend metformin as the first-line drug to be used to treat patients with type 2 diabetes mellitus.

Second, the finding of a more remarkable risk reduction in the PP analyses than in the ITT analyses (Table 2, Table 3 and Table 4) implied that adherence to metformin treatment may provide more remarkable protection against MM.

Third, metformin is an inexpensive drug, safe and without the risk of hypoglycemia when used as a monotherapy. Therefore, repurposing metformin as a preventive agent or an adjuvant therapeutic agent for MM is worthy of more in-depth investigation.

Fourth, two-thirds of patients with MM may have cardiac events [56], and metformin may exert a prophylactic effect on cardiotoxicity induced by carfilzomib [57] and may have a positive impact on the life expectancy of patients with MM and heart failure [58]. In our previous studies, we also demonstrated a reduced risk of hypertension [59], atrial fibrillation [60] and heart failure [61] among metformin users. Therefore, metformin may exert a protective effect on cardiovascular diseases in the absence or presence of MM.

### 4.5. Strengths

There are some merits to this study. First, we can be more confident in generalizing the findings because of the use of a nationwide database that covers >99% of the population.

Second, the risk of self-reporting bias could be avoided because of the use of existing medical records.

Third, different socioeconomic statuses may lead to a serious problem of detection bias in other countries. However, this would not be the case in our healthcare system. Cancer is considered a catastrophic illness, and most medical copayments can be waived for patients with a certified diagnosis of cancer. Additionally, many medical expenses can be waived for veterans and patients with low incomes or receiving drug refills for chronic disease.

### 4.6. Limitations

There are some limitations. First, we did not have information on radiation exposure for adjustment. We tried to balance radiation by using ocular pterygium as a surrogate diagnosis for exposure to UV sunlight (Table 1). Because the standardized difference of ocular pterygium was <10%, potential confounding from radiation might be minimal.

Second, obesity is a well-recognized risk factor for MM [6], but we did not have anthropometric data of body height and body weight in the database for analyses. Although we used a diagnosis of obesity rather than an actual measurement of body height and body weight in the analyses, the prevalence rates of obesity in metformin (−) and metformin (+) subjects were 1.88% and 4.38%, respectively (Table 1). In our earlier epidemiologic survey, the prevalence rates of obesity in diabetes patients defined by a body mass index of ≥25 and ≥30 kg/m^2^ were 33.5% and 7.1%, respectively [62]. Therefore, a diagnosis of obesity might only have been labelled in patients with severe obesity, and the use of an ICD-9-CM diagnosis of obesity might have underestimated the true prevalence rates of obesity. It is worth pointing out that metformin is always recommended for obese patients, and this was truly reflected by the higher prevalence of such a diagnosis among metformin initiators (Table 1). The higher prevalence of obesity among metformin (+) subjects would only have underestimated the true beneficial effect of metformin on MM.

Third, statins [63,64] and aspirin [65,66] exhibit anti-cancer activity in MM cells. Although the distribution of aspirin between metformin (+) and metformin (−) was balanced, more patients were using statins in the metformin (+) group (33.11% versus 25.92%, Table 1). This imbalance in the use of statins might have exerted a residual confounding even though we had weighted the hazard ratios by PS. To further confirm that the lower risk of MM among the metformin (+) group would not be impacted by the use of statins, we additionally conducted a sensitivity analysis after excluding those who had used any statin. The respective hazard ratios comparing the metformin (+) to the metformin (−) groups were 0.653 (95% confidence interval: 0.532–0.800) in the ITT analysis and 0.350 (95% confidence interval: 0.257–0.478) in the PP analysis. The consistency of the results supported the robustness of the findings.

Fourth, MM is an insidious disease with asymptomatic premalignant stages [1,2]. If the delayed diagnosis of MM differed significantly between the metformin (+) and metformin (−) groups, this might have caused a biased estimate. Therefore, more future studies are required to investigate the possible roles of other confounders.

Fifth, because MM related to genetic mutations are not studied, their potential confounding could not be excluded. However, if the MM-related genetic mutations did not distribute differentially between the metformin (−) and the metformin (+) groups, it was expected that the estimated hazard ratios would only bias toward the null.

Sixth, we did not have the pathology of bone marrow biopsies and/or aspiration for confirmation of MM diagnosis and for additional analyses.

### 4.7. Conclusions

Patients with type 2 diabetes mellitus who have been initiated with metformin therapy have a significantly lower risk of MM, especially when they adhere to the treatment.

## Figures and Tables

**Table 1:** Characteristics of metformin initiators (metformin (+)) and non-metformin initiators (metformin (−)).

Variables	Metformin (−)		Metformin (+)		Standardized Difference
	(n = 242,305)		(n = 497,248)		
	n	%	n	%	
**Basic data**					
Age * (years)	58.53	13.24	55.85	13.27	−7.97
Sex (men)	130,885	54.02	268,493	54.00	0.23
Time without antidiabetic drugs after diabetes diagnosis * (years)	1.69	1.49	1.90	1.63	16.81
Occupation					
I	90,856	37.50	201,375	40.50	
II	47,896	19.77	201,375	40.50	5.00
III	58,895	24.31	98,440	19.80	−10.60
IV	44,658	18.43	91,520	18.41	−1.77
Living region					
Taipei	75,424	31.13	172,251	34.64	
Northern	27,863	11.50	64,724	13.02	5.31
Central	43,151	17.81	88,404	17.78	−1.40
Southern	43,090	17.78	76,419	15.37	−6.31
Kao-Ping and Eastern	52,777	21.78	95,450	19.20	−7.32
**Major comorbidities**					
Hypertension	151,453	62.51	311,678	62.68	3.41
Dyslipidemia	112,992	46.63	280,842	56.48	22.43
Obesity	4554	1.88	21,773	4.38	13.84
**Diabetes-related complications**					
Nephropathy	38,439	15.86	68,263	13.73	−4.86
Eye diseases	10,265	4.24	35,298	7.10	12.61
Stroke	47,950	19.79	89,808	18.06	−2.98
Ischemic heart disease	76,125	31.42	149,648	30.10	−0.97
Peripheral arterial disease	28,382	11.71	28,382	5.71	2.41
Hypoglycemia	32,891	13.57	4075	0.82	−0.60
**Medications that are commonly used by diabetes patients**					
Angiotensin-converting enzyme inhibitor/angiotensin receptor blocker	117,006	48.29	244,835	49.24	4.23
Calcium channel blocker	117,036	48.30	221,799	44.61	−5.48
Statin	62,806	25.92	164,644	33.11	17.33
Fibrate	53,605	22.12	120,126	24.16	6.27
Aspirin	94,028	38.81	198,040	39.83	4.14
**Comorbidities that might affect exposure or outcome**					
Chronic obstructive pulmonary disease	87,069	35.93	181,570	36.51	2.88
Tobacco abuse	2515	1.04	8632	1.74	6.19
Alcohol-related diagnoses	10,955	4.52	23,674	4.76	1.85
Heart failure	30,372	12.53	30,372	6.11	−4.77
Parkinson’s disease	4903	2.02	8158	1.64	−2.57
Dementia	12,636	5.21	21,669	4.36	−3.62
Head injury	1795	0.74	5291	1.06	3.72
Valvular heart disease	17,158	7.08	32,891	6.61	−1.36
Helicobacter pylori infection	895	0.37	895	0.18	3.08
Epstein–Barr virus infection	1239	0.51	2687	0.54	0.66
Hepatitis B virus infection	2758	1.14	8533	1.72	5.19
Hepatitis C virus infection	6971	2.88	14,303	2.88	0.57
Human immunodeficiency virus disease	126	0.05	262	0.05	0.14
Cirrhosis of liver without mention of alcohol	9737	4.02	15,689	3.16	−4.18
Other chronic nonalcoholic liver disease	15,042	6.21	37,844	7.61	6.18
Autoimmune diseases	14,130	5.83	31,922	6.42	3.25
Organ transplantation	643	0.27	776	0.16	−2.17
Insomnia	41,568	17.16	95,610	19.23	6.92
Malaise and fatigue	6584	2.72	19,867	4.00	7.75
History of some disorders of the central nervous system	40,853	16.86	87,193	17.54	2.84
Immunosuppression	9893	4.08	16,989	3.42	−3.00
Benign neoplasm of bone and articular cartilage	671	0.28	1689	0.34	1.21
Bone fractures	37,975	15.67	83,546	16.80	3.99
Ocular pterygium	7482	3.09	15,316	3.08	0.65
Disorders of the thyroid gland	16,356	6.75	44,442	8.94	8.82
Nutritional deficiencies	5725	2.36	10,176	2.05	−1.93
Depression	12,424	5.13	29,444	5.92	4.10
Cancer	22,305	9.21	43,716	8.79	−0.78

* “Age” and “Time without antidiabetic drugs after diabetes diagnosis” are expressed as mean and standard deviation.

**Table 2:** Incidence of multiple myeloma and hazard ratios comparing metformin initiators (metformin (+)) to non-metformin initiators (metformin (−)) in the intention-to-treat and per-protocol analyses.

Metformin Initiation	Incident Case Number	Cases Followed	Person-Year	Incidence Rate (per 100,000 Person-Years)	Hazard Ratio	95% Confidence Interval	*p*-Value
Intention-to-treat							
Metformin (−)	220	242,305	1,534,914.14	14.33	1.000		
Metformin (+)	260	497,248	2,608,969.26	9.97	0.710	(0.593–0.850)	0.0002
Per-protocol							
Metformin (−)	106	242,305	758,110.62	13.98	1.000		
Metformin (+)	106	425,726	2,064,133.42	5.14	0.355	(0.270–0.466)	<0.0001

**Table 3:** Subgroup analyses by age and sex.

Subgroup	Incident Case Number	Cases Followed	Hazard Ratio	95% Confidence Interval	*p*-Value
**(1) Men**					
Intention-to-treat					
Metformin (−)	114	130,885	1.000		
Metformin (+)	146	268,493	0.762	(0.596–0.975)	0.0306
Per-protocol					
Metformin (−)	53	130,885	1.000		
Metformin (+)	62	229,825	0.425	(0.294–0.616)	<0.0001
**(2) Women**					
Intention-to-treat					
Metformin (−)	106	111,420	1.000		
Metformin (+)	114	228,755	0.653	(0.501–0.851)	0.0016
Per-protocol					
Metformin (−)	53	111,420	1.000		
Metformin (+)	44	195,901	0.286	(0.191–0.429)	<0.0001
**(3) Age** **≥ 60 years**					
Intention-to-treat					
Metformin (−)	160	109,977	1.000		
Metformin (+)	181	184,338	0.810	(0.655–1.003)	0.0533
Per-protocol					
Metformin (−)	80	109,977	1.000		
Metformin (+)	69	151,698	0.429	(0.310–0.594)	<0.0001
**(4) Age < 60 years**					
Intention-to-treat					
Metformin (−)	60	132,328	1.000		
Metformin (+)	79	312,910	0.715	(0.510–1.002)	0.0512
Per-protocol					
Metformin (−)	26	132,328	1.000		
Metformin (+)	37	274,028	0.403	(0.242–0.671)	0.0005

**Table 4:** Sensitivity analyses.

Models	Incident Case Number	Cases Followed	Hazard Ratio	95% Confidence Interval	*p*-Value
**1. Excluding two consecutive prescriptions of metformin spanning more than 6 months**					
Intention-to-treat					
Metformin (−)	220	242,305	1.000		
Metformin (+)	115	246,249	0.795	(0.633–1.000)	0.0500
Per-protocol					
Metformin (−)	106	242,305	1.000		
Metformin (+)	27	177,989	0.285	(0.187–0.435)	<0.0001
**2. Excluding patients who happened to be treated with incretin-based therapies during follow-up**					
Intention-to-treat					
Metformin (−)	211	210,110	1.000		
Metformin (+)	245	416,396	0.726	(0.604–0.87)	0.0007
Per-protocol					
Metformin (−)	106	210,110	1.000		
Metformin (+)	98	347,493	0.384	(0.291–0.507)	<0.0001
**3. Excluding patients who had ever been treated with thiazolidinediones**					
Intention-to-treat					
Metformin (−)	186	183,856	1.000		
Metformin (+)	217	364,689	0.745	(0.612–0.907)	0.0034
Per-protocol					
Metformin (−)	102	183,856	1.000		
Metformin (+)	86	298,735	0.401	(0.301–0.534)	<0.0001
**4. Excluding patients with a diagnosis of any cancer other than multiple myeloma during follow-up**					
Intention-to-treat					
Metformin (−)	220	194,986	1.000		
Metformin (+)	260	415,629	0.692	(0.578–0.829)	<0.0001
Per-protocol					
Metformin (−)	106	194,986	1.000		
Metformin (+)	106	358,038	0.338	(0.258–0.444)	<0.0001

## Data Availability

Public availability of the dataset is restricted by local regulations to protect privacy.

## Supplementary Materials

The following supporting information can be downloaded at: https://www.mdpi.com/article/10.3390/cancers14225637/s1, Table S1: The disease diagnoses and their corresponding codes of the International Classification of Diseases, Ninth Revision, Clinical Modification (ICD-9-CM) used in the study.
