# Supplementary material for: The Risk of Multiple Myeloma Is Reduced in Metformin Initiators: A Retrospective Cohort Study in Taiwanese Patients with Type 2 Diabetes Mellitus
Source: Cancers (Basel). 2022 Nov 17;14(22):5637. doi: 10.3390/cancers14225637 (PMC9688273; doi:10.3390/cancers14225637)
Supplement: Supplementary file 1 [file cancers-14-05637-s001.zip › cancers-2006308-supplementary.pdf]

Supplementary Table S1. The disease diagnoses and their corresponding codes of the International Classification of Diseases, Ninth Revision, Clinical Modification (ICD-9-CM) used in the study.

Disease diagnoses	ICD-9-CM codes
Diabetes mellitus	250.XX
Multiple myeloma	203.0
Hypertension	401–405
Dyslipidemia	272.0–272.4
Obesity	278
Nephropathy	580–589
Eye diseases	250.5: diabetes with ophthalmic manifestations<br>362.0: diabetic retinopathy<br>369: blindness and low vision<br>366.41: diabetic cataract<br>365.44: glaucoma associated with systemic syndromes
Stroke	430–438
Ischemic heart disease	410–414
Peripheral arterial disease	250.7, 785.4, 443.81, 440–448
Hypoglycemia	251.0, 251.1, 251.2
Chronic obstructive pulmonary disease	490–496
Tobacco abuse	305.1, 649.0, 989.84
Alcohol-related diagnoses	291, 303, 535.3, 571.0–571.3, 980.0
Heart failure	398.91, 402.11, 402.91, 404.11, 404.13, 404.91, 404.93, 428
Parkinson's disease	332
Dementia	abridged codes of A210 or A222, or as ICD-9-CM codes of 290.0, 290.1, 290.2, 290.4, 294.1, 331.0–331.2, 331.7–331.9
Head injury	959.01
Valvular heart disease	394–396, 424, 746
Helicobacter pylori infection	041.86
Epstein-Barr virus infection	075, 710.3, 710.4
Hepatitis B virus infection	070.22, 070.23, 070.32, 070.33, V02.61
Hepatitis C virus infection	070.41, 070.44, 070.51, 070.54, V02.62
Human immunodeficiency virus disease	042
Cirrhosis of liver without mention of alcohol	571.5
Other chronic nonalcoholic liver disease	571.8

Autoimmune diseases	579.0: celiac disease<br>696.0: psoriatic arthritis<br>psoriasis: 696.1<br>710.0: systemic lupus erythematosus<br>710.1: systemic sclerosis<br>710.2: Sjogren's syndrome<br>714.0: rheumatoid arthritis
Organ transplantation	V42
Insomnia	780.52
Malaise and fatigue	780.79
History of some disorders of the central nervous system	340–349
Benign neoplasm of bone and articular cartilage	213.0–213.9
Bone fractures	800–829
Ocular pterygium	372.40-372.44
Disorders of thyroid gland	240–246
Nutritional deficiencies	260–269
Depression	296 and 311
Cancer	140–208
